# PPP1R14B as a potential biomarker for the identification of diagnosis and prognosis affecting tumor immunity, proliferation and migration in prostate cancer

**DOI:** 10.7150/jca.101100

**Published:** 2024-10-21

**Authors:** Yizhong Bao, Yixiu Ni, Aokang Zhang, Jun Chen

**Affiliations:** 1 Zhejiang Provincial Key Lab of Geriatrics & Geriatrics Institute of Zhejiang Province, Department of Geriatrics, Zhejiang Hospital, Hangzhou, 310013, Zhejiang Province, China.; 2 School of Medicine, Zhejiang University, Hangzhou, 310013, Zhejiang Province, China.

**Keywords:** prostate cancer, bioinformatics, biomarkers, tumor immunity, anti-PCa drugs

## Abstract

Prostate cancer (PCa) is a malignancy that affects men and is characterized by metastasis and high rates of morbidity. The objective of this study was to explore novel PCa biomarker with potential diagnostic and therapeutic value and relationships between it and tumor immunity and development. A total of 32 key genes were screened out via LASSO based upon 188 intersection genes obtained from WGCNA and DEGs analysis in GSE32571, and PPP1R14B was further identified by COX regression based on the TCGA database and validated by qRT-PCR. Although it has been reported that PPP1R14B may have a certain correlation with the prognosis of uterine corpus endometrial carcinoma, breast cancer and gastrointestinal cancer, there are none of studies about correlation between PPP1R14B and PCa. Predictive ability analysis showed that PPP1R14B had greatly predictive values in occurrence and prognosis of PCa. Immune analysis revealed that overexpression of PPP1R14B was related to the increase of ALKBH2, UCK2, RAC3 and RAB17 and the decrease of CD40, DKK3, COL17A1 and PGRMC1, which would result in downregulation of plasma cells, upregulation of T regulatory cells and disorder of macrophage proportion to suppress adaptive immune directed against PCa. GSEA analysis showed that PPP1R14B, as an inhibitor of PP1, its overexpression was mainly involved in regulating pathways associated with MYC, E2F, PFN1 and so on, which was participated in the regulation of immune factors such as CD40, RAC3, COL17A, DKK3, as well as biological processes such as proliferation and migration. Patients with higher PPP1R14B expression responded more sensitively to drugs selumetinib and vorinostat, zebularine, azacitidine and VER155008. In summary, PPP1R14B was a potential diagnostic and prognostic biomarker of PCa and its high expression had closely association with tumor immune inhibition, proliferation and migration, providing a new target for drug therapy and immunotherapy in PCa.

## Introduction

The prostate is a part of the male reproductive system, located at the bottom of the bladder and surrounding the urethra. The prostate is one of the important male organs and prostate fluid provides nutrition and protection for sperm, helping to maintain their survival and motility. Prostate cancer is one of the most common cancers among men worldwide. Recent data showed that PCa accounts for 26% of new male malignant tumor cases, ranking first in incidence rate, and it ranks second in the mortality rate of male malignant tumors 1, 2. Early-stage PCa can be controlled through treatments such as radiation therapy and surgery. However, as PCa progresses and invades, early-stage PCa can develop into more aggressive metastatic prostate cancer (mPC) with poor prognosis 3. The main treatment method for metastatic hormone sensitive prostate cancer (mHSPC), which is one of the subtypes of mPC, is androgen deprivation therapy (ADT). However, relevant studies have shown that ADT has a risk of inducing cardiovascular disease 4, 5. For another subtype of mPC, metastatic castration resistant prostate cancer (mCRPC), abiraterone and docetaxel are commonly used as their main treatment drugs 6, 7. However, resistance to these drugs is inevitable during the treatment process, and research reports have shown that cross resistance to different drugs may occur during the treatment of mCRPC 8, 9. Although there have been some advancements in the treatment of PCa, the current options for treating mPC are still inadequate, which made it become a significant medical issue for men. Therefore, it is very important to further study the molecular mechanism of PCa occurrence and provide new diagnostic and therapeutic biomarkers for PCa.

In this study, based on gene expression data and clinical sample information obtained from Gene Expression Omnibus (GEO) and the Cancer Genome Atlas (TCGA) databases, we used weighted Gene Co-expression Network Analysis (WGCNA), least absolute shrinkage and selection operator (LASSO) logistic regression and COX proportional hazards regression analysis to screen for biomarker which has strong correlation with PCa. qRT-PCR was employed to validate for the expression difference of biomarker from prostate cells with/without PCa. Subsequently, COX regression analysis, nomograms, Receiver Operating Characteristic Curve (ROC) and Decision Curve Analysis (DCA) were used to validate biomarker to obtain the correlation between biomarker and the occurrence, survival, and prognosis of PCa. In addition, immune infiltration analysis is also used to explore the relationship between biomarker and immune cells, immune checkpoints, and immune chemokines, aiming to gain a deeper understanding of the immune mechanisms involved in the development of PCa. Furthermore, GSEA analysis was performed on biomarker to understand their functions. What's more, we conducted drug sensitivity analysis to identify effective drugs that may have a positive impact on PCa. Finally, the competitive endogenous RNA (ceRNA) network was constructed to describe the regulatory mechanism of biomarkers in PCa.

## Materials and Methods

### Data acquisition and analysis

**Figure 1:**
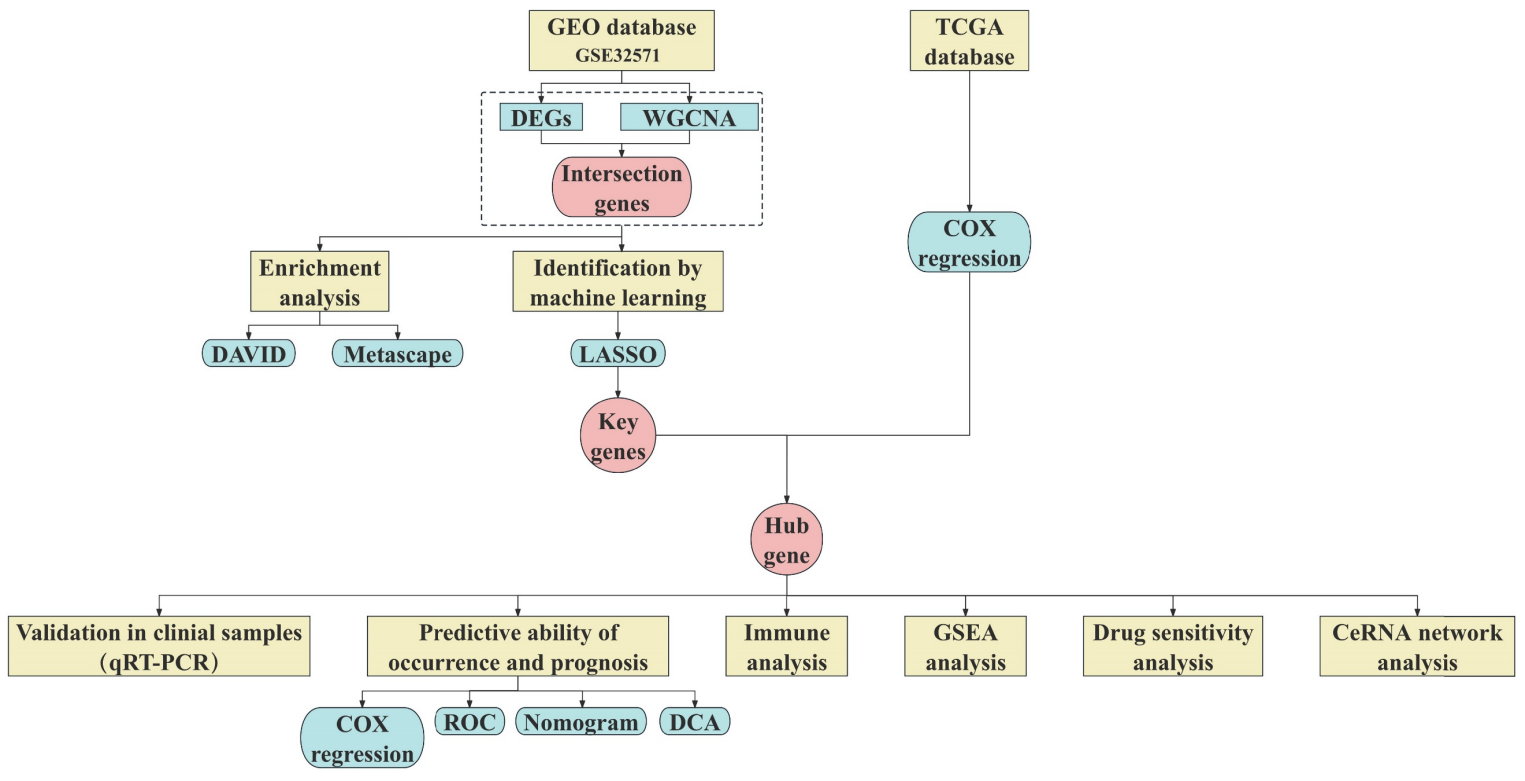
Overview of the study design.

The research process of this study was shown in **Fig. 1**. The Series Matrix File of GSE32571 was downloaded from the GEO database (https://www.ncbi.nlm.nih.gov/geo/) using the keyword "prostate cancer". GSE32571 is a dataset based on gene signatures in PCa linked to the Gleason score, containing a total of 98 samples (59 tumor samples and 39 normal samples). Inclusion criteria for tumor samples considered: (i) all patients were male and diagnosed with pathologically confirmed PCa; (ii) Gleason score is 6-10; (iii) Primary tumor tissue undergoing radical prostatectomy. Exclusion criteria include (i) excluding samples from patients with a prior history of malignancy; (ii) Exclude samples from patients with multiple malignant tumors present simultaneously; (iii) Exclude samples from patients with metastatic tumors. The clinical information table of the samples can be found in **Supplementary Table 1**.

After analyzing and processing the data based on a p-value less than 0.05 and an absolute value of log fold change (|logFC|) greater than 1, we screened differentially expressed genes (DEGs) from 98 samples. In addition, volcano plots and heatmaps were created to visualize the differential expression of DEGs using the aforementioned data.

Furthermore, TCGA database (https://portal.gdc.cancer.gov/) was accessed to obtain the clinical information of 361 PCa samples and 52 normal samples. The inclusion and exclusion criteria for these tumor samples are consistent with GSE32571. The clinical information table of the samples can be found in **Supplementary Table 2**.

### WGCNA analysis

Genes in GSE 32571 were analyzed using WGCNA to explore critical module genes. The Pearson correlation coefficients between genes were calculated based on GSE32571. The scale-free network was constructed and the appropriate threshold was selected for node network construction. Using two-step construction, after the adjacency matrix was transformed into a topological overlap matrix, the clustering tree was generated through hierarchical clustering. Furthermore, clustering was combined through a dynamic cut to obtain co-expression modules. The correlation between modules and traits was measured, and modules with a correlation greater than 0.6 were considered critical modules. Based on DEGs and critical module genes, a Venn diagram was established on bioinformatics to screen intersection genes.

### Enrichment analysis

Intersection genes were used for Gene Ontology (GO) enrichment analyses (including cellular components (CCs), molecular functions (MFs), and biological pathways (BPs)) and Kyoto Encyclopedia of Genes and Genomes (KEGG). Statistical analysis was performed using the DAVID (https://david.ncifcrf.gov/) and graphical charting was utilized by the clusterProfiler R package. We subsequently performed a functional enrichment analysis by the Metascape (http://metascape.org).

### Acquisition of hub gene

Machine learning is a breakthrough technology that is flexibly applied in the collection and processing of large amounts of data. It involves learning, identifying, and grasping associations within the data through the analysis of vast amounts of data. Utilizing these learned insights, machine learning enables prediction, classification, optimization, and decision-making 10. In this study, LASSO was employed to further select key genes related to PCa among the intersection genes. The "glmnet" package in R was utilized for LASSO logistic regression analysis.

The COX regression model is based on the concept of hazard ratio, which can estimate the risk ratio of the occurrence of an event. It is usually used for survival analysis and prediction of individual survival time. In this study, the "survival" package of R language was used to perform univariate COX regression analysis on selected data from the TCGA database to screen out the hub gene.

### Validation of hub gene by qRT-PCR

qRT-PCR was performed to confirm the expression of hub gene in the PCa and normal control (NC) groups. Human PCa epithelial cell lines (PC-3) and normal prostate epithelial cell lines (RWPE-1) were purchased from the Institute of Biochemistry and Cell Biology of the Chinese Academy of Sciences (Shanghai, China) (**Supplementary Fig. 1**). PC-3 cells are originated from a 62-year-old white male with grade IV primary prostate cancer (Gleason score > 6). Media for cell culture and fetal bovine serum (FBS) were obtained from GIBCO. The PC-3 and RWPE-1 cell lines were cultured in Ham's F12 nutrient medium (Invitrogen). RPMI 1640 medium (Invitrogen) were supplemented with 10% FBS (Sigma) and keratinocyte serum-free medium (Invitrogen), respectively. All the cells were cultured at 37 ℃ in an atmosphere of 5% CO_2_. All dissected samples were immediately stored in liquid nitrogen until they were prepared for total RNA extraction.

Briefly, total RNA was extracted from the PCa and NC groups using TRIZOL reagent (Life Technologies, Gaithersburg, MD, USA) according to the manufacturer's instructions. To detect the purity of the RNA, the OD values were measured using a NanoDrop (NanoDrop One, Thermo Fisher Scientific, Waltham, MA, USA). Reverse transcription was performed on each RNA sample (500 μg) using the PrimeScript RT reagent kit with a gDNA eraser (Takara Bio, Dalian, China) according to the manufacturer's instructions. For qRT-PCR analysis, the reaction mixture (including cDNA, DEPC water, forward primers, and reverse primers) was run in an iQ5 real-time PCR machine (Bio-Rad, Hercules, CA, USA) according to the instructions for SYBR Premix Ex Taq II (Takara Bio, Dalian, China). All the expression levels were normalized to those of the internal control (β-actin). The qRT-PCR cycles were as follows: step1, preparative denaturation (30 s at 95 ℃); step 2, 40 cycles of denaturation (5 s at 95 ℃) and annealing (30 s at 60 ℃); and step 3, dissociation, following the manufacturer's protocol. Forward primer of hub gene was as followed: GCTGGGTTCCTCCGAGGTA; reverse primer of hub gene was as followed: GAGTTGAAAGAGGGTGTGAGAG. The 2^-ΔΔct^ method was used to calculate relative gene expression levels.

### Correlation between hub gene and PCa

To further validate significance of hub gene in PCa, correlations between hub gene and PCa were investigated with TCGA and GEO datasets. Differentially expression of hub gene was analyzed and visualized using the DESeq2 R package from TCGA database. Based on GSE 32571, the ROC curves of hub gene and known PCa-related genes GDF15 and AMACR and area under the curve (AUC) were plotted by the pROC R package. Nomogram was designed using the rms R package according to GEO datasets to construct the risk profile model of PCa occurrence based on hub gene. Calibration curve and DCA were used to evaluate the predictive accuracy and reliability of the nomogram. Further, according to COX regression analysis, the risk score of the hub gene is obtained, and its survival curve is then plotted using the "survminer" R package.

### Correlation between PCa and hub gene integrating clinical features

In order to explore the correlation between PCa and hub gene binding clinical features, we conducted univariate and multivariate COX regression analyses on hub gene and 5 clinical features using the "survival" package using TCGA datasets, respectively. Further, the prognostic nomogram was constructed by integrating hub gene expression and clinical factors. Moreover, ROC curves, calibration curves and DCA in both training and validation cohorts were performed to assess the accuracy and discrimination of the nomogram.

### Immune infiltration

To analysis immune infiltration of PCa and control groups, we used the GEO datasets to calculate the infiltration proportion of 22 kinds of immune cells with the CIBERSORT algorithm and analyzed differences with a significance threshold of p < 0.05. What's more, correlations between hub gene and immmune cells, checkpoints and chemokines were studied by the “dplyr” and “ggplot2” packages.

### GSEA analysis

GSEA (http://software.broadinstitute.org/gsea/index.jsp) is a powerful tool that can be used to reveal the biological significance of gene expression data, and it can also help researchers identify key biological functions and pathways under different conditions. Based on the hub gene expression data obtained from GEO, GSEA was used to conduct pathway enrichment analysis for hub gene high-expression and low-expression samples respectively. |NES|> 1, p < 0.05 and FDR < 0.25 were considered significant enrichment.

### Drug Sensitivity Analysis

The Cancer Therapeutics Response Portal (CTRP) data and GEO datasets were utilized to calculate the sensitivity of common anticancer drugs targeting PCa and hub gene according to the half maximal inhibitory concentration (IC50) using the “oncoPredic” package.

### Construction of ceRNA network

To better understand the mechanism of how lncRNA regulates gene expression through miRNA, the ceRNA network was constructed based on the miRWalk (http://mirwalk.umm.uni-heidelberg.de), TargetScan (http://www.targetscan.org/) and miRNet (https://www.mirnet.ca/miRNet/home.xhtml) databases. We predicted the miRNAs of hub gene using miRWalk and TargetScan databases, and obtained intersection results from the two databases. Next, the miRNet database was used to predict potential lncRNAs of overlapping miRNAs. Subsequently, a ceRNA regulatory network was constructed by integrating the interaction between miRNA and lncRNA.

### Statistical analysis

All statistical analyses were conducted by R language (version 4.3.1). The Kaplan Meier method was used for survival analysis. The prognostic potential of hub genes was studied using univariate and multivariate COX models. A significance level of P<0.05 is considered statistically significant.

## Results

### Attainment of intersection genes

**Figure 2:**
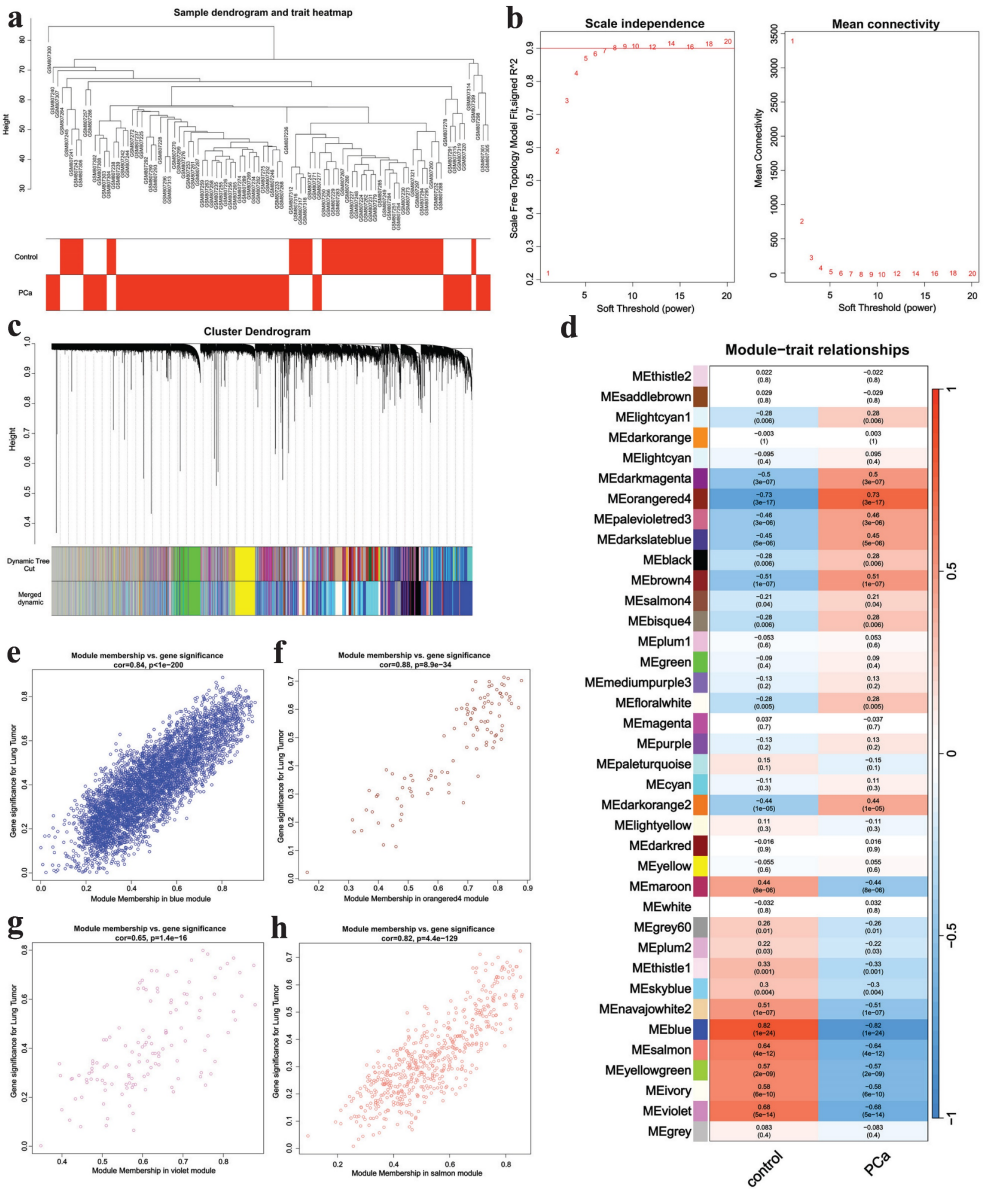
WGCNA analysis results.** a** The samples clustering tree, **b** network topology analysis, **c** clustering tree, **d** module heatmap, **e** scatter plot of blue module, **f** scatter plot of orange4 module, **g** scatter plot of violet module, **h** scatter plot of salmon module.

WGCNA was applied to analyze genes in GSE32571. A sample clustering tree was obtained in **Fig. 2a**. Then we chose the soft-threshold 8 according to **Fig. 2b**. Finally, 38 modules were identified (**Fig. 2c**) and we ascertained four critical modules including the blue module (cor= 0.82, p = 1e^-24^), the orange4 module (cor = 0.73, p = 3e^-17^), the violet module (cor = 0.68, p = 5e^-14^) and the salmon module (cor = 0.64, p = 4e^-12^) (**Fig. 2d**). The orange4 module had a positive correlation with PCa while the other three modules showed negative relationship with this disease. The scatter plots of critical modules were shown in **Fig. 2e-h**.

**Figure 3:**
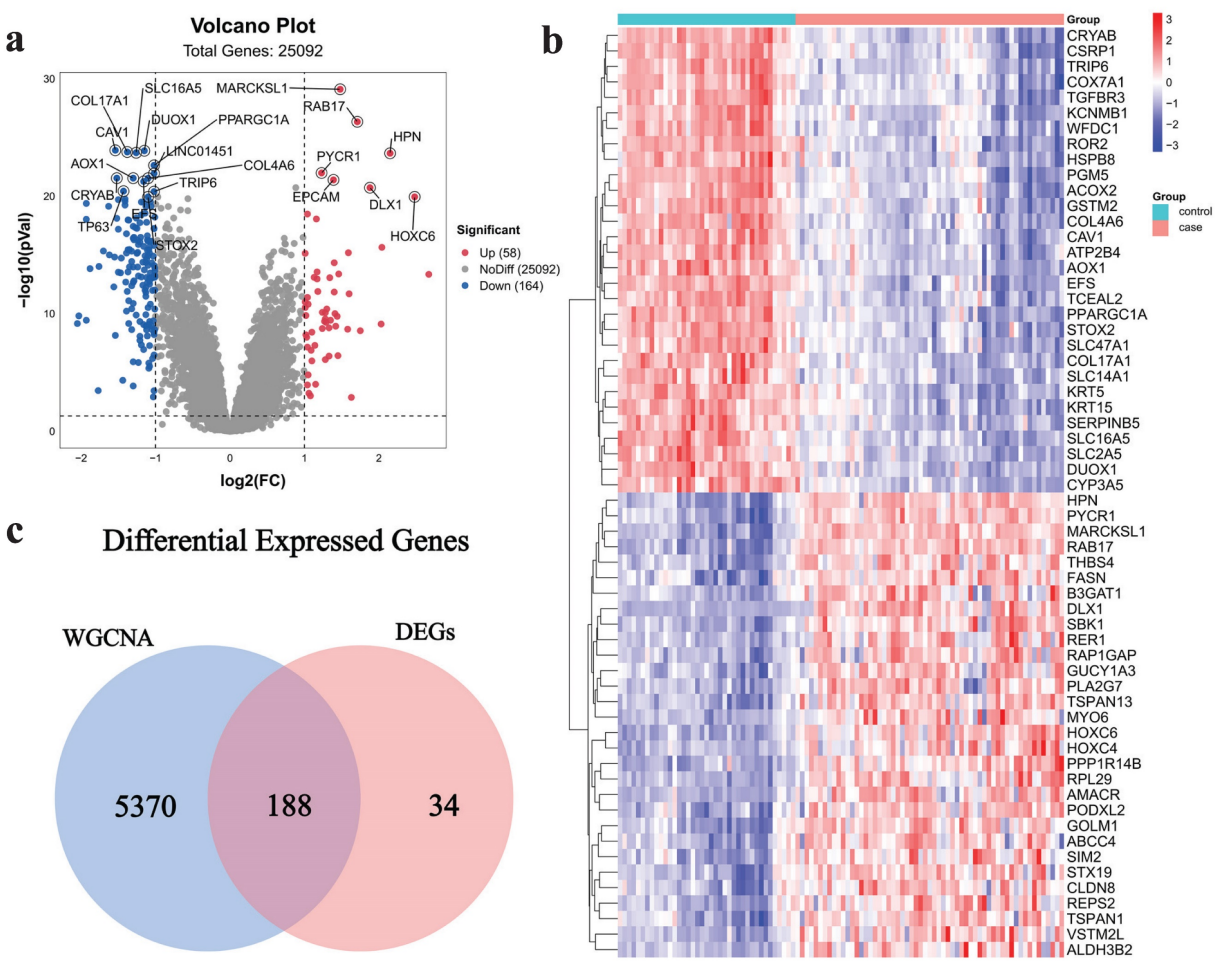
Acquisition of intersection genes.** a** Volcano map of DEGs, **b** heatmap of DEGs, **c** Venn diagram of critical module genes and DEGs.

222 DEGs (including 58 up-regulated genes and 164 down-regulated genes) were acquired from the dataset GSE32571, which was obtained from the GEO database. The volcano plot (**Fig. 3a**) and heat map (**Fig. 3b**) were used to visualize the DEGs. The intersection of critical module genes and 222 DEGs yielded 188 intersection genes (**Fig. 3c**).

### Enrichment analysis

**Figure 4:**
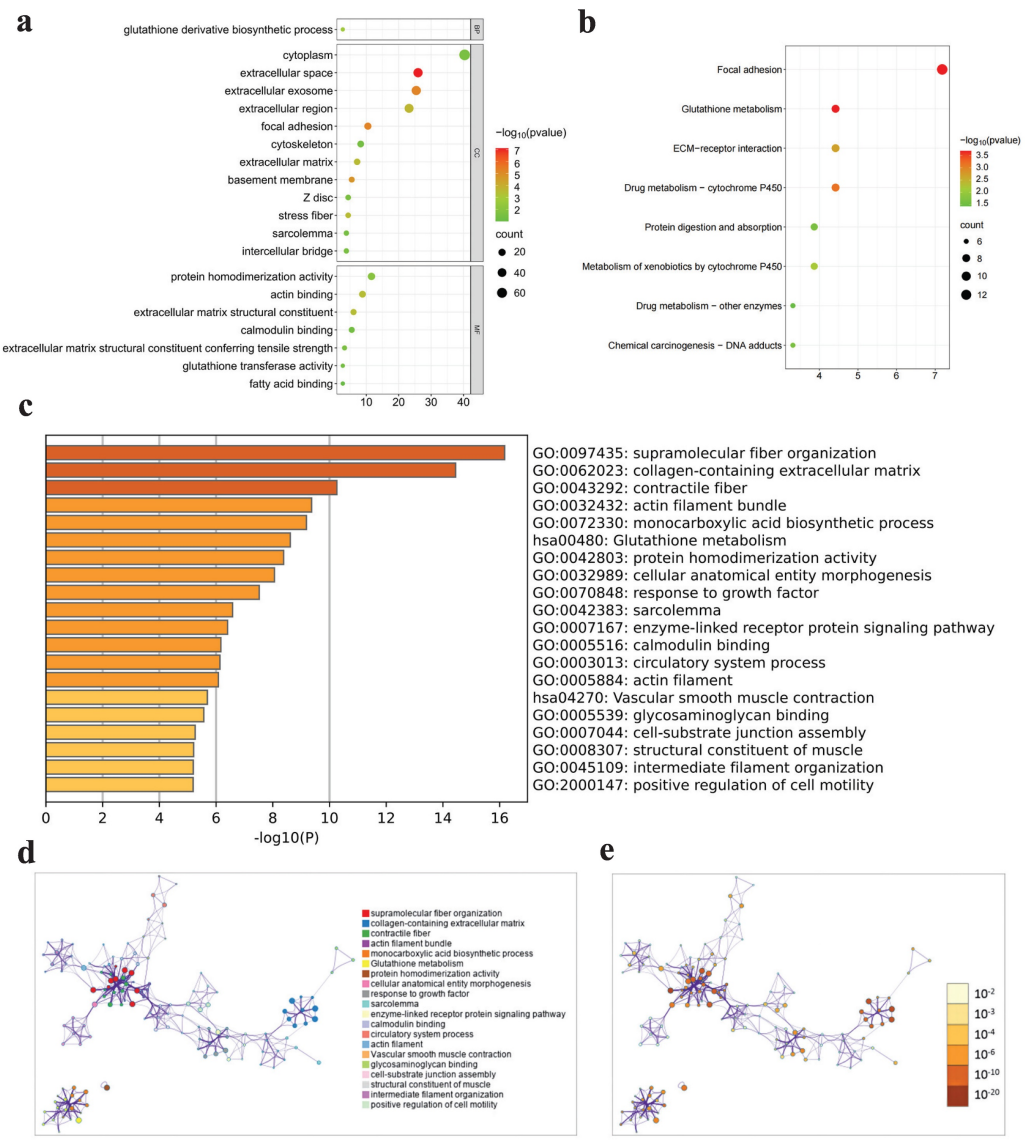
Enrichment analysis results.** a** GO enrichment for three terms, **b** KEGG enrichment, **c-e** Metascape enrichment.

GO, KEGG and Metascape analyses were performed to investigate the functional mechanisms of 188 intersection genes. A total of 188 intersection genes were enriched in (i) CCs: extracellular space, extracellular exosome and focal adhesion and so on; (ii) MFs: extracellular matrix structural constituent, actin binding and protein homodimerization activity and so on; and (iii) BPs: glutathione derivative biosynthetic process and so on (**Fig. 4a**). The results of KEGG pathway enrichment exhibited that enriched pathways mainly contained focal adhesion, glutathione metabolism and drug metabolism - cytochrome P450 and so on (**Fig. 4b**). The results of Metascape enrichment analysis showed that these intersection genes were mainly enriched in functions and pathways such as supramolecular fiber organization, glutathione metabolism and protein homodimerization activity and so on (**Fig. 4c-e**).

### Acquisition and validation of hub gene

**Figure 5:**
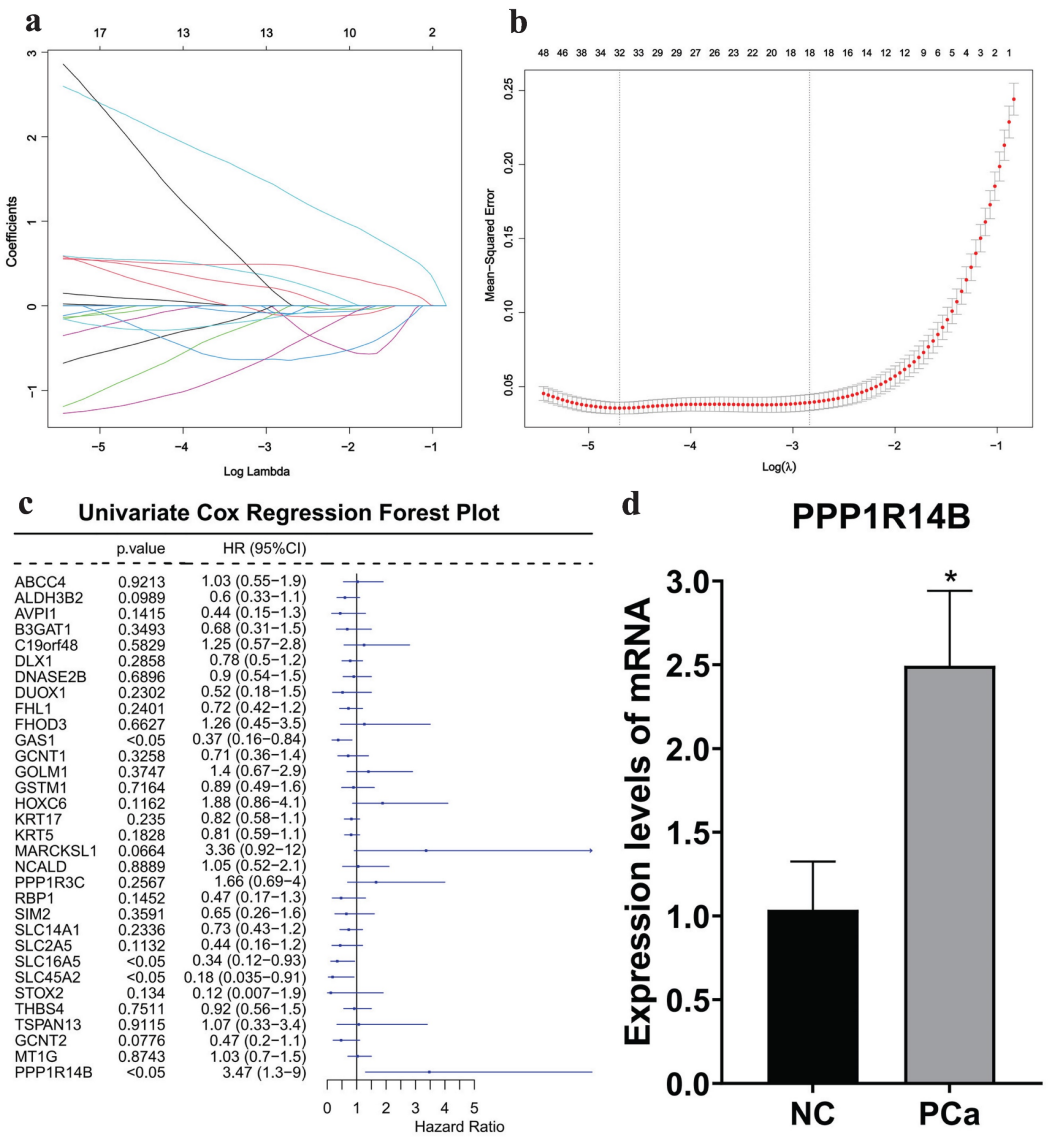
Acquisition and validation of hub gene. **a-b** LASSO regression analysis results, **c** COX regression analysis results, **d** qRT-PCR results in cells with/without PCa.

Then, based on the 188 intersection genes, a LASSO regression model was constructed with the optimal λ value of 0.00911853 to further screen key genes. We applied LASSO regression analysis to select 32 predictive genes as key genes (**Fig. 5a-b**).

Furthermore, based on the clinical information obtained from the TCGA database, we conducted a univariate COX regression analysis on 32 key genes. The forest plot showed that the p-value of PPP1R14B was less than 0.05, which was statistically significant, and it had the highest HR value of 3.47 among the 32 genes (**Fig. 5c**). qRT-PCR showed that the expression of PPP1R14B (**Fig. 5d**) in the PCa group (**Supplementary Fig. 1a**) was significantly higher than that in the NC group (**Supplementary Fig. 1b**). Therefore, PPP1R14B is considered the hub gene of this study.

### Correlation between hub gene and PCa

**Figure 6:**
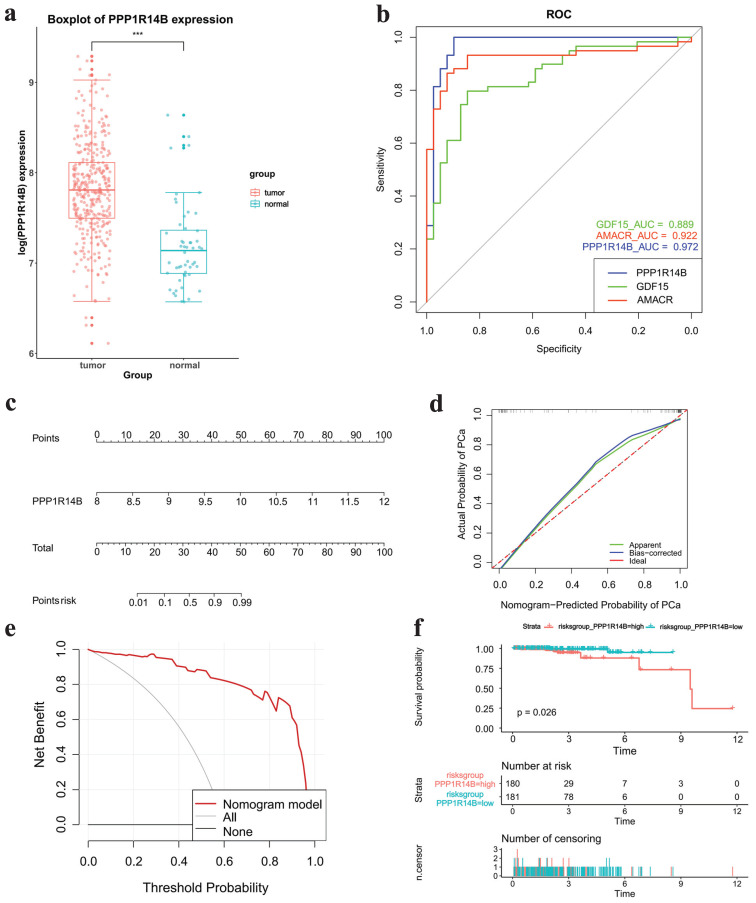
Correlation between Hub Gene and PCa. **a** Boxplot of hub gene expression in different groups in TCGA datasets, **b** ROC analysis results of hub gene, GDF15 and AMACR, **c-e** nomogram, calibration curve and DCA results of hub gene for PCa occurrence, **f** survival curve of hub gene.

The boxplot showed that hub gene PPPP1R14B was more greatly expressed in tumor tissues than in normal tissues based on TCGA datasets (**Fig. 6a**). ROC analysis indicated that PPP1R14B and two known PCa-related genes GDF15 and AMACR had AUC values > 0.8, with PPP1R14B having the largest AUC value (AUC, 0.972), AMACR the middle (AUC, 0.922) and GDF15 the smallest (AUC, 0.889) (**Fig. 6b**). Nomogram prediction models, calibration curve and DCA showed that PPP1R1B was a high-risk factor in the occurrence of PCa (**Fig. 6c-e**). The survival curve of PPPP1R14B drawn according to the risk score showed that the survival probability of the low-risk group was higher when the survival time was the same (**Fig. 6f**).

### Correlation between PCa and hub gene integrating clinical features

**Figure 7:**
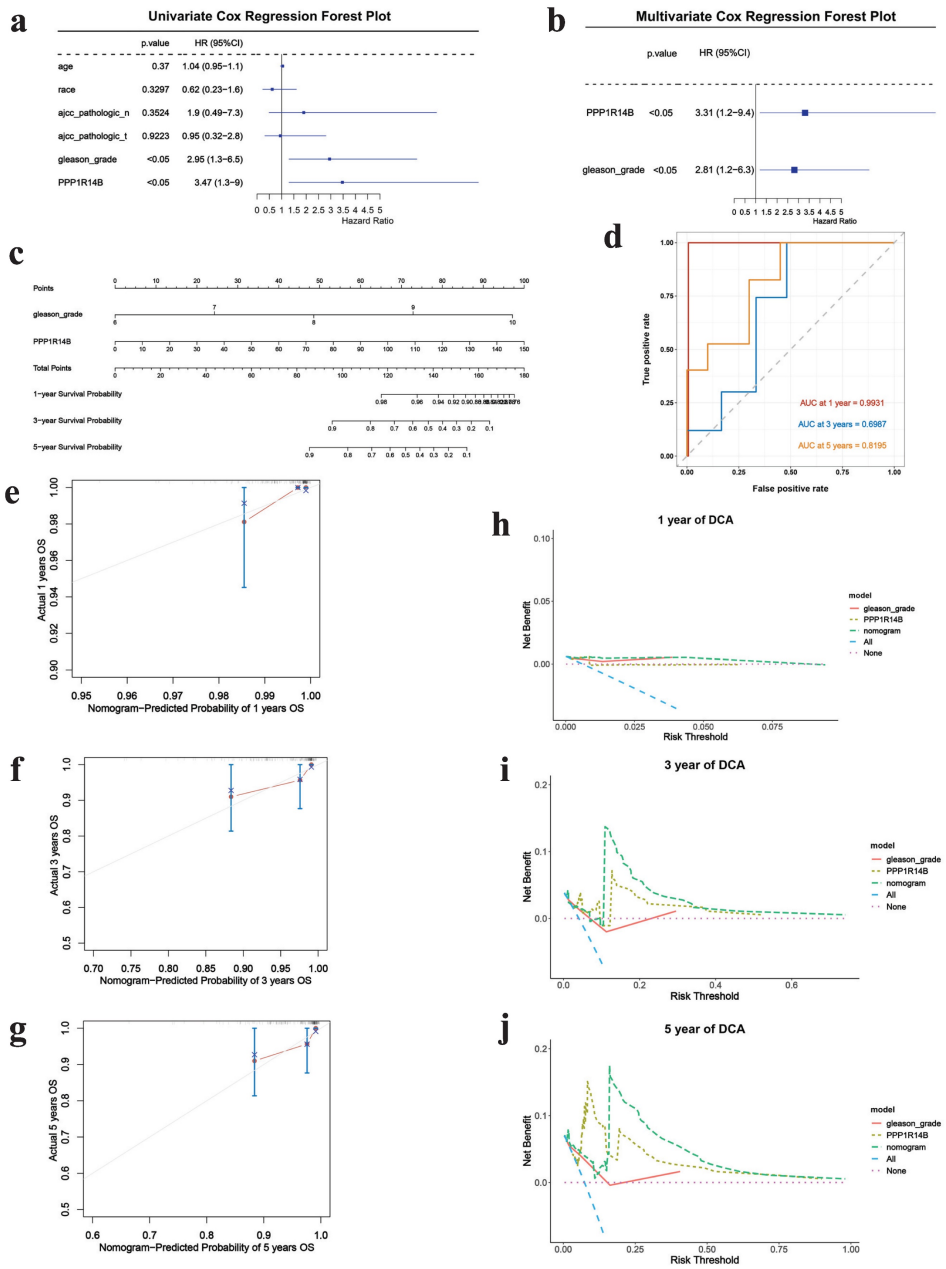
Correlation between PCa and hub gene integrating clinical features based on the training cohort. **a** Univariate COX regression results, **b** multivariate COX analysis results, **c** prognostic nomogram to predict 1-, 3-, and 5-year overall survival, **d** ROC for 1-, 3- and 5-year overall survival, **e-g** calibration curves of prognostic nomogram for 1-, 3- and 5-year overall survival, **h-j** DCA of prognostic nomogram, hub gene and gleason-grade for 1-, 3- and 5-year overall survival.

Next, we further explored the impact of PPP1R14B combined with clinical factors according to TCGA datasets. The screening results of univariate COX regression analysis showed gleason grade (p<0.05, HR: 2.95, 95% CI: 1.3-6.5) (**Fig. 7a**). Subsequently, multivariate COX analysis was performed by combining gleason grade with PPP1R14B, and the results revealed that PPP1R14B (p<0.05, HR: 3.31, 95% CI: 1.2-9.4) and gleason grade (p<0.05, HR: 2.81, 95% CI: 12.2-6.3) (**Fig. 7b**) had independent prognostic value for overall survival of PCa.

**Figure 8:**
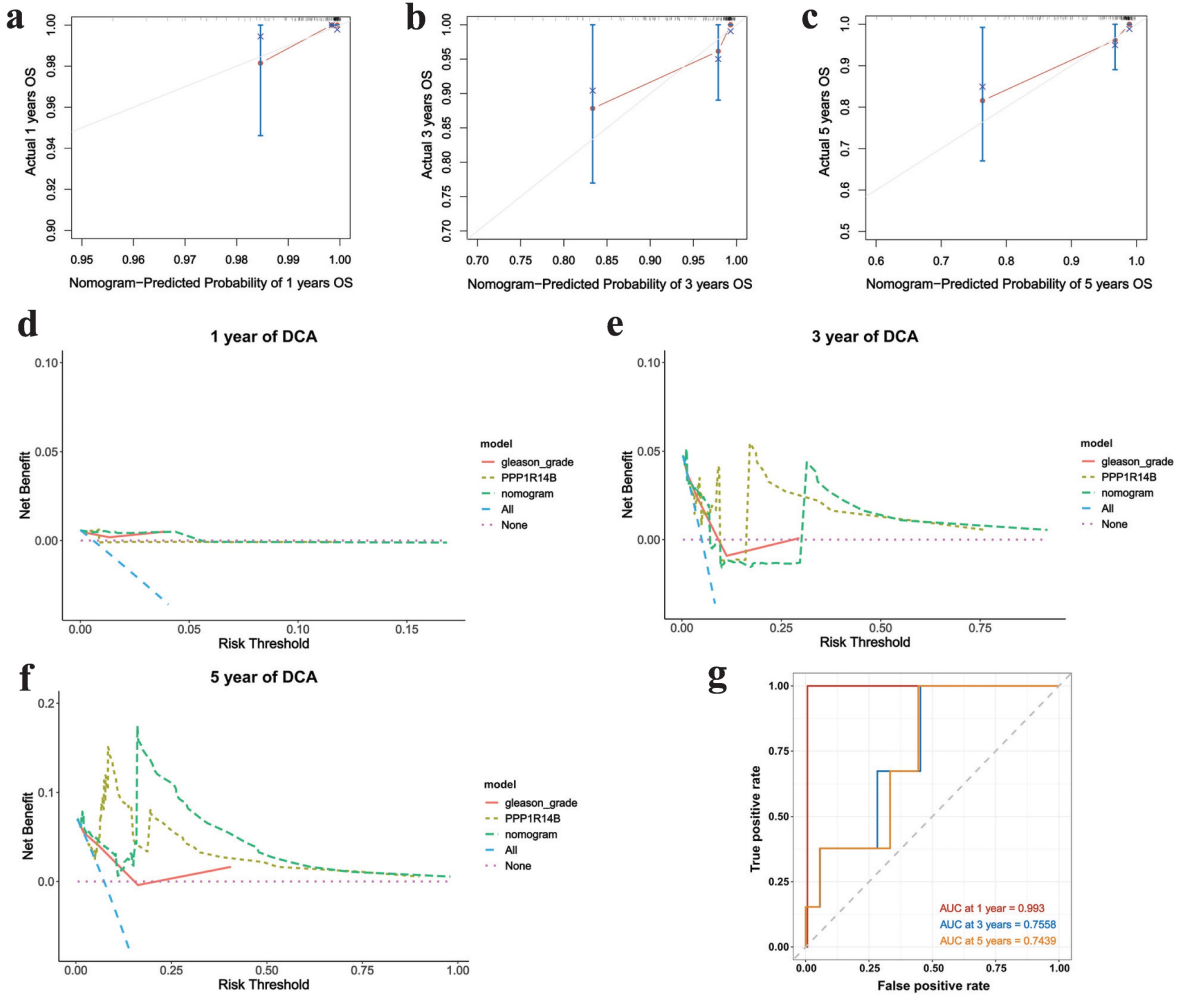
Correlation between PCa and hub gene integrating clinical features based on the validation cohort. **a-c** Calibration curves of prognostic nomogram for 1-, 3- and 5-year overall survival, **d-f** DCA of prognostic nomogram, hub gene and gleason-grade for 1-, 3- and 5-year overall survival, **g** ROC for 1-, 3- and 5-year overall survival.

The nomogram was used to study the predictive prognostic value of hub gene PPP1R14B expression combining with clinical features and gleason - grade, showing that high PPP1R14B expression and gleason-grade had shorter overall survival (**Fig. 7c**). The predicted accuracy of the nomogram in both the training and validation cohorts was evaluated by the ROC curve, shown the different AUC values (training cohort: 1-year = 0.9931, 3-year = 0.6987, 5-year = 0.8195; validation cohort: 1-year = 0.993, 3-year = 0.7558, 5-year = 0.7439) (**Fig. 7d**,** Fig. 8g**). The calibration curves showed that the risks predicted by the nomogram were highly consistent with the observed overall survival for 1, 3, and 5 years in both cohorts (**Fig. 7e-g**, **Fig. 8a-c**). Moreover, DCA reflecting the performance of the prediction model confirmed that PPP1R14B expression could greatly predict overall survival and PPP1R14B combining with gleason grade had even better prediction ability in two cohorts (**Fig. 7h-j**, **Fig. 8d-f**).

### Immune analysis

**Figure 9:**
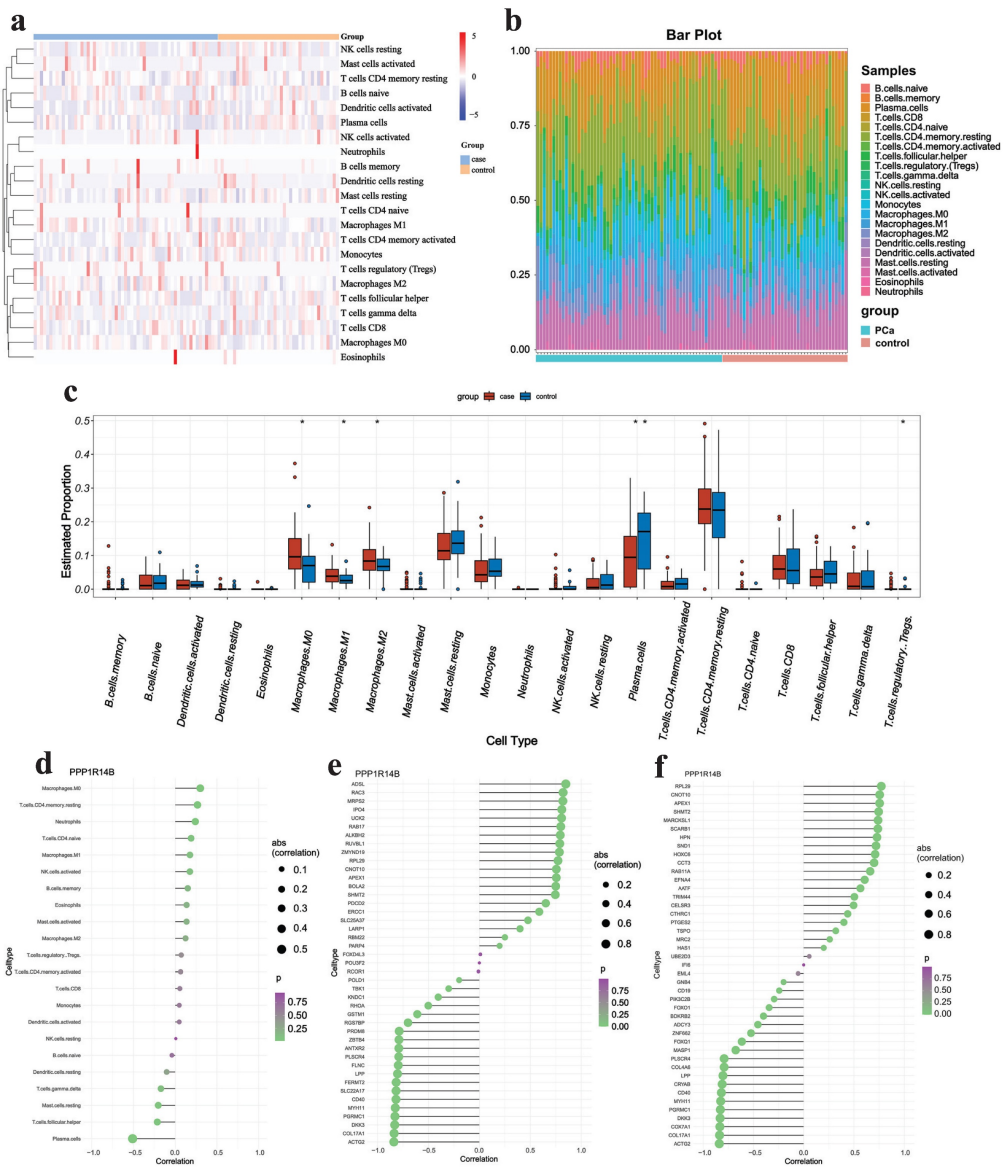
Immune analysis results.** a-b** Immune infiltrating cells estimated by CIBERSORT algorithm, **c** boxplot displaying differences in scores for infiltrating immune cells, **d-f** lollipop plot showing the correlation between hub gene and immune cells, checkpoints and chemokines.

We first focused on the abundance of 22 types of immune cells using the CIBERSORT algorithm (**Fig. 9a&b**). The results showed that PCa owned a higher abundance of M0 macrophage, M1 macrophage, M2 macrophage and T regulatory cells (Tregs) but had a lower abundance of plasma cells (**Fig. 9c**). In addition, the correlation analysis indicated that hub gene PPP1R14B had weak positive correlation (r < 0.4) with M0 macrophage, T cells CD4 memory resting and neutrophils but negatively correlated with plasma cells (|r| > 0.4) and was lowly negatively related (|r| < 0.4) to T cells follicular helper and mast cells resting (**Fig. 9d**). It was further confirmed that PPP1R14B was strongly positively correlated (r > 0.7) with immune checkpoints in ADSL, RAC3, MRPS2, IPO4, UCK2 and so on, and greatly negatively correlated (r < -0.7) with them in ACTG2, COL17A1, DKK3, PGRMC1, MYH11, CD40, SLC22A17, FERMT2, LPP and so on (**Fig. 9e**). What's more, the association between this hub gene and immune chemokine showed that PPP1R14B had stiff positive relevance (r > 0.7) to RPL29, CNOT10, APEX1, SHMT2, MARCKSL1, SCARB1, HPN, SND1, HOXC6, CCT3 and so on while the hub gene had hard negative correlation (r < -0.7) with ACTG2, COL17A1, COX7A1, DKK3, PGRMC1, MYH11, CD40, CRYAB, LPP and so on (**Fig. 9f**).

### GSEA analysis

**Figure 10:**
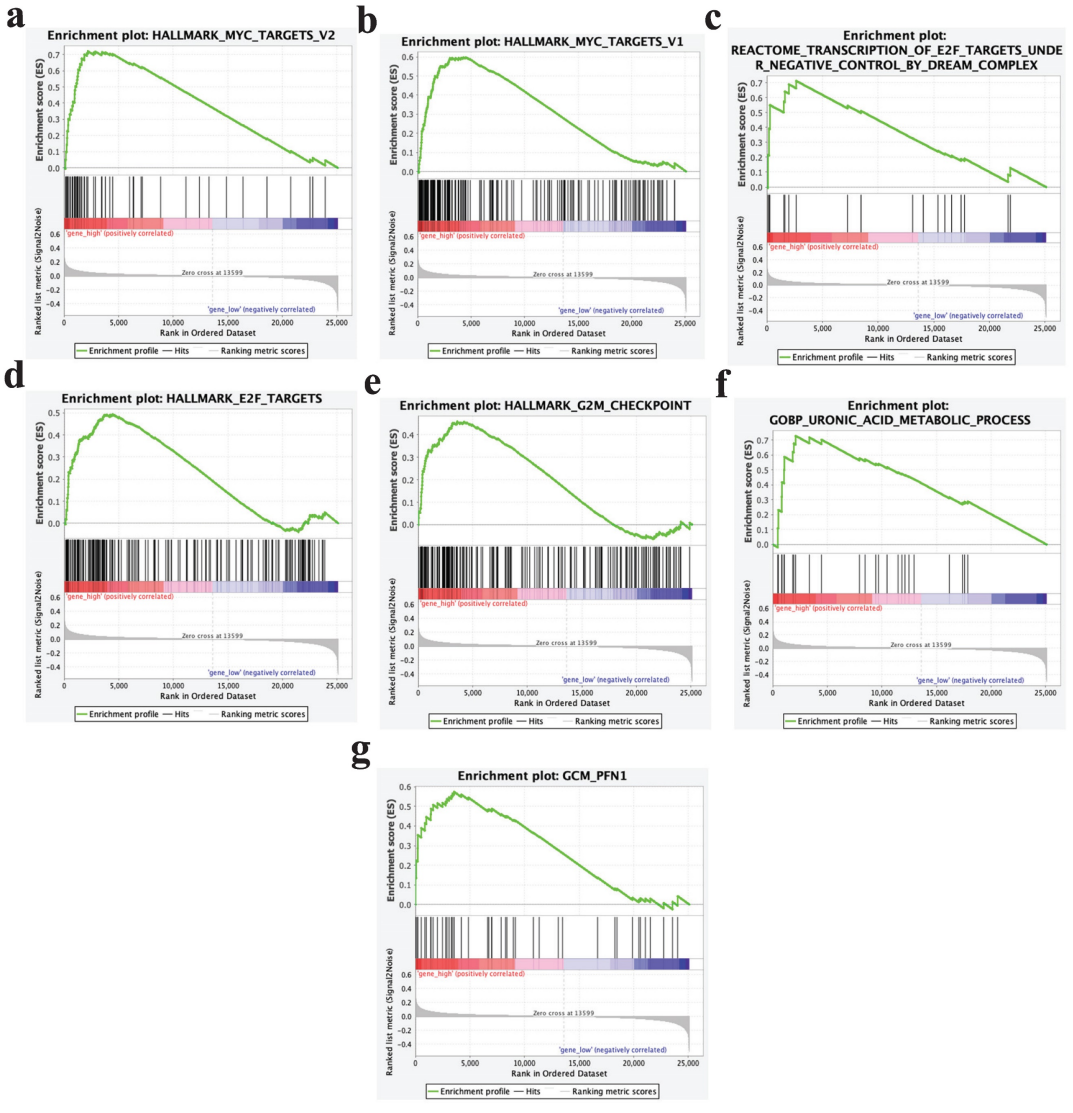
GSEA analysis results.** a** Myc targets V2, **b** Myc targets V1, **c** transcription of E2F targets under negative control by dream complex, **d** E2F targets, **e** G2M checkpoint, **f** uronic acid metabolic process, **g** GCM- PFN1.

GSEA analysis results showed that samples with high expression of PPP1R14B could significantly enrich pathways such as Myc targets V2, Myc targets V1, transcription of E2F targets under negative control by dream complex, E2F targets, G2M checkpoint, uronic acid metabolic process, GCM- PFN1 and so on (**Fig. 10a-g**).

### Drug sensitivity analysis

**Figure 11:**
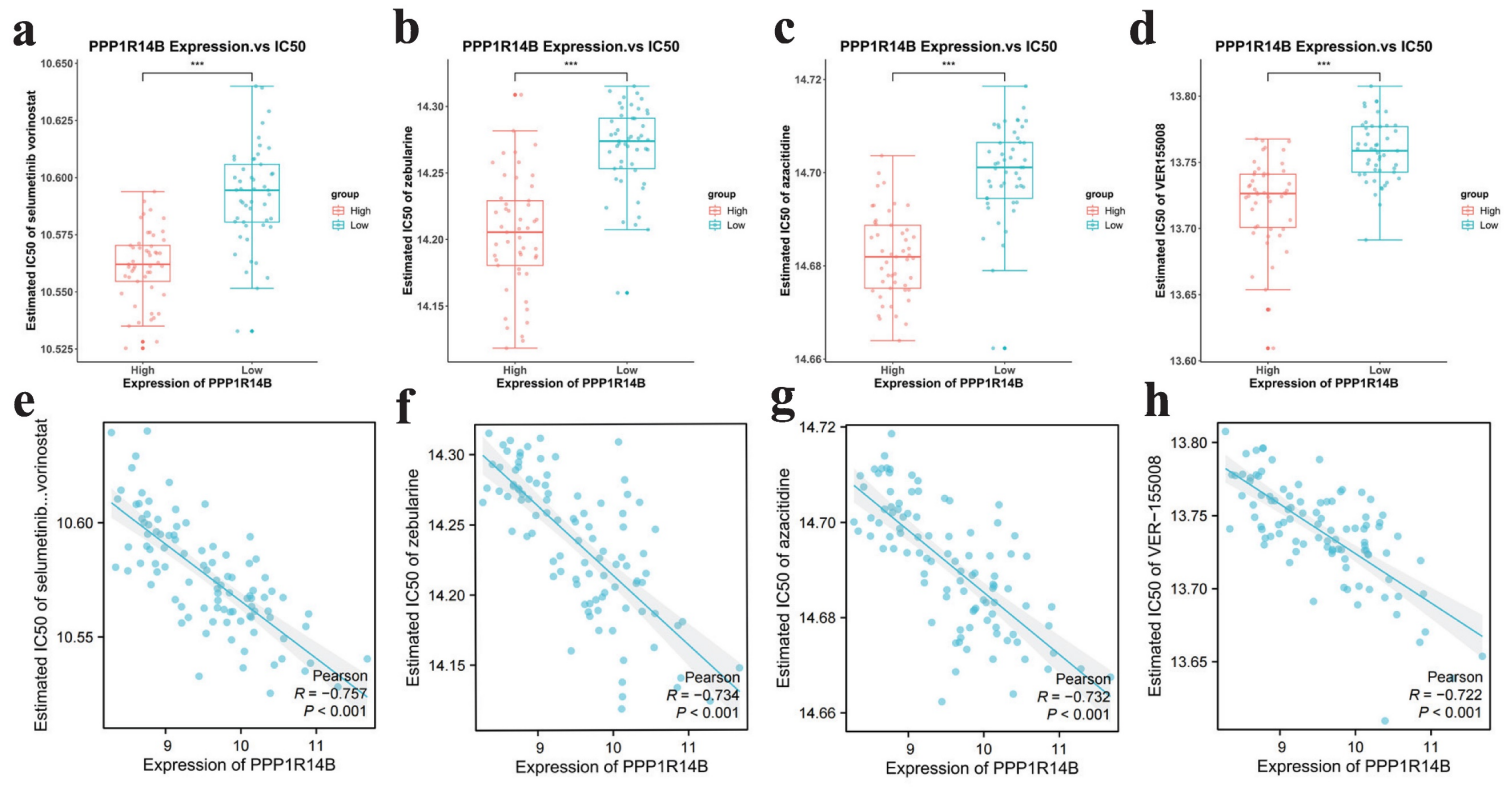
Drug sensitivity results targeting hub gene.** a-d** IC50 of selumetinib and vorinostat, zebularine, azacitidine and VER155008 in low and high expression groups of hub gene, **e-h** scatter plots between expression of hub gene and IC50 of selumetinib and vorinostat, zebularine, azaacitidine and VER155008.

To explore the possibility of applying hub gene PPP1R14B to the personalized and accurate treatment of PCa, we analyzed sensitivity of different anticancer drugs according to the IC50 value. The results showed that 4 commonly used drugs selumetinib vorinostat, zebularine, azacitidine and VER155008 targeting PPP1R14B showed satisfactory differences in sensitivity, that is, patients in the high-risk group were more likely to be sensitive to them (**Fig. 11a-h**).

### Construction of ceRNA Network

**Figure 12:**
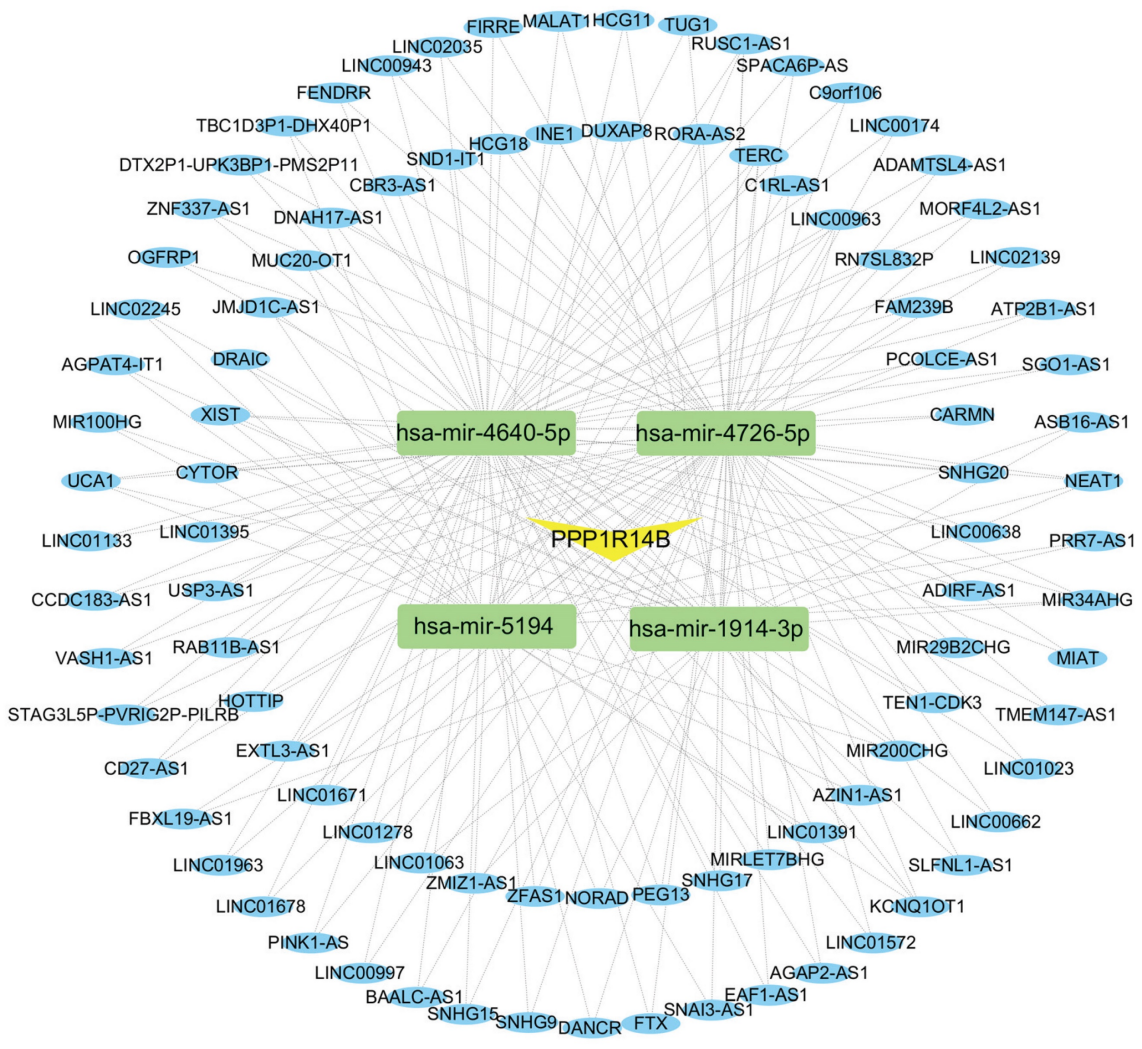
CeRNA network built with miRWalk, TargetScan and miRNet. Every box stands for a miRNA, and each circle represents a lncRNA.

The ceRNA regulatory network was constructed to investigate the regulatory mechanism of PPP1R14B. We used miRWalk and TargetScan databases to predict miRNAs associated with PPP1R14B and obtained 21 miRNAs from these two databases. Then, based on the obtained miRNAs, we searched for relevant lncRNAs in the miRNet database. Among the 21 miRNAs, 4 miRNAs (hsa-miR-1914-3p, hsa-miR-4640-5p, hsa-miR-4726-5p, and hsa-miR-5194) were found to be associated with 94 lncRNAs, and a total of 204 miRNA-lncRNA pairs were obtained (**Fig. 12**).

## Discussion

PCa is the second most diagnosed solid-organ cancer, after lung cancer, in men 11. PCa has the complicated pathogenesis, and is easy to metastasize, which is challenging to treat. The available prostate-specific antigen (PSA)-based diagnostic and target-based therapeutic strategies approaches have come up with various false positives and off-target side effects in medical diagnosis and therapeutics of PCa 12. As a result, exploring the pathogenesis and identifying the biomarker of PCa is highly critical to therapy. In this study, GEO gene expression datasets were employed by carrying out DEG, WGCNA and LASSO analyses, and furtherly hub gene was screened by COX regression based on TCGA datasets and validated through qRT-PCR in PCa and normal cells. Nomograms, calibration curves, DCA, ROC analysis and COX regression were used to explore relationship between PCa and hub gene, as well as correlation between PCa and hub gene binding clinical features. We delved potential mechanism of hub gene in PCa using GSEA, immune analysis and ceRNA networks. Finally, through the drug sensitivity analysis, we screened therapeutic drugs targeting hub gene related to PCa.

Totally, 188 intersection genes were screened out according to GSE 32571 via differentially expression and WGCNA analyses, and significantly enriched in extracellular matrix structural constituent, glutathione derivative biosynthetic process, protein homodimerization activity, focal adhesion and so on. Extracellular matrix structural constituent and immune cell are important parts of the distinct tumor microenvironment (TME) of PCa, which take part in tumor proliferation and progression of PCa 13. Glutathione is an antioxidant that acts as a free radical scavenger and a detoxifying agent in cells, and useful in a multitude of biological processes including cellular division, differentiation and proliferation and so on, and preserves sufficient levels of cysteine and detoxifies xenobiotics, which can promote the survival and proliferation of tumor cells and also confer resistance to cancer cells 14. Protein homodimerization activity can increase the proliferation ability of cancer cells, reduce cell apoptosis, and promote invasion and metastasis 15. Focal adhesion can regulate cell proliferation, survival and migration, and promote tumor growth 16. In summary, the enrichment results revealed that 188 intersection genes had close relationship with PCa.

Based on intersection genes, 32 key genes were further screened through LASSO analysis. Furthermore, among them, we found that PPP1R14B had a remarkable relationship with PCa. Both qRT-PCR and TCGA datasets indicated that hub gene PPP1R14B highly expressed in PCa groups. GEO datasets showed that PPP1R14B had area under the curve (AUC) value > 0.95 that were more than known PCa-related genes AMACR and GDF15, displaying ideal predictive performance for PCa occurrence, as assessed by the ROC curves, and the nomogram models based on hub gene expression level further confirmed this point. Subsequently, COX univariate regression analysis was performed based on clinical sample information obtained from the TCGA database. The hazard ratio (HR: 3.47, 95% CI: 1.3-9, p<0.05) and survival curve of PPP1R14B both reveal that PPP1R14B is a high-risk factor affecting the occurrence and prognosis of PCa.

Furthermore, we continued to explore the impact of PPP1R14B combined with clinical factors on PCa. Through univariate COX regression analysis, gleason grade was identified as a clinical factor significantly affecting the prognosis of PCa. Subsequently, multivariate COX regression analysis was conducted based on PPP1R14B combined with gleason grade, and the results showed that both PPP1R14B and gleason grade have a strong correlation with the prognosis of PCa and play an important role in the occurrence and development of PCa. Subsequently, we established the prognostic nomogram of PPP1R14B and gleason grade based on TCGA datasets and verified its excellent prognostic predictive performance through calibration curves and DCA in training and validation cohorts. The results additionally showed that PPP1R14B could function as a vital prognostic factor in PCa.

PCa is a multifactorial and complex disease involving several immunological, environmental, physiologic, and genetic factors 9, so we explored the relationships between PCa and immune infiltration. In our study, PCa owned a higher proportion of M0 macrophage, M1 macrophage, M2 macrophage and Tregs but relatively lower ones of plasma cells. Mononuclear cells are recruited to the TME by stromal cells and tumor-secreted chemokines and growth factors secreted by the tumor to differentiate into M0 macrophages 17. M0 macrophages display two phenotypes, namely M1 and M2. M0 polarization is driven toward the M1 phenotype by miR-203, where M1 macrophages actively present antigens to effector cells in the immune system and secreted multiple pro-inflammatory factors, thereby influencing inflammatory responses and anti-tumor immunity during tumor progression 18. M2 macrophages in tumor tissue release cytokines epidermal growth factor and transforming growth factor β1 (TGF-β1), which facilitate tumor cell division, proliferation, and growth 18. Thus, an increase in the abundance of M1 macrophage could promote inflammatory response and anti-PCa effects, thereby promoting PCa cell apoptosis, but an increase in the proportion of M2 macrophage would inhibit these processes. In previous vivo and vitro studies have shown that Tregs are another type of immune cell that suppress natural killer (NK) cells and CD8^+^ T lymphocytes by secreting inhibitory cytokines (such as transforming growth factor β, TGF β) 19, which is directly related to a greater likelihood of death from PCa 20. Plasma cell is a kind immune cell that can generate and secret antibody 21. When the proportion of plasma cells decreased, the production of antibodies might be limited, which would lead to impaired immune response against prostate cancer. In summary, our results revealed that inhibition of anti-tumor immunity in PCa was closely associated with the increase of macrophage and Tregs and the decrease of plasma cells, which might lead to improvement of PCa cells anti-apoptosis, activity inhibition of NK cells and CD8+ T cells, and strength frustration of humoral immunity.

Furthermore, we explored the correlation between hub gene PPP1R14B and the TME. We found that PPP1R14B had highly negative correlation with immunologic factors CD40, COL17A1, DKK3 and PGRMC1, but had strongly positive relevance to ALKBH2, UCK2, RAC3 and RAB17. CD40, a member of the tumor necrosis factor receptor family, is expressed by B cells, professional antigen-presenting cells, as well as non-immune cells and tumors 22. CD40 signaling of B cells promotes germinal center formation, immunoglobulin (Ig) isotype switching, somatic hypermutation of the Ig to enhance affinity for antigen, and finally the formation of long-lived plasma cells and memory B cells 23. Tests on mice show that in B cells, suppression of ALKBH2 activates NF-κB 24 that is one of the transcription factors of CD40 and promotes the transcription and expression of CD40 25, which can enhance the degree of differentiation of B cells into plasma cells and promote antibody synthesis. It was demonstrated that downregulation of UCK2 also activated the TNF α/NF-κB signaling pathway 26. Our results revealed that the high expression of PPP1R14B might lead to a decrease in CD40 through direct regulation or possible ALKBH2/UCK2-NF-κB-CD40 signaling, thereby hindering differentiation of B cells and formation of plasma cells, which resulted in insufficient expression of antibodies and strength frustration of humoral immunity directed against PCa. Additionally, research suggested that DKK3 could upregulate the antitumor immune function of CD56^bright^ NK cells by inducing its differentiation and improving its cytotoxicity 27. The reduced expression of DKK3 can stimulate TGF-β signaling 28, which can help naïve T cells differentiate to Tregs 20, thereby preventing NK and CD8+ T cells from exerting anti-tumor effects 19. UCK2, uridine-cytidine kinase 2, is a key regulator of pyrimidine metabolism, and the increased expression of UCK2 partly stimulated TGF β1, which also inhibited NK cell infiltration and NK-cell-mediated killing 26. When the expression level of PPP1R14B increased, it might cause the downregulation of DKK3 and the upregulation of UCK2, which would facilitate TGF β signaling and further Tregs generation, thereby reducing the cytotoxicity of NK cells and promoting CD8+ T cells exhaustion to inhibit cellular immunity in PCa. What's more, RAC3, a member of the Rac family of small guanosine triphosphatases (GTPase), regulates a variety of cell functions, including the organization of the cytoskeleton, cell migration, and invasion 29, and macrophage morphological plasticity and migration is Rac GTPase signalling dependent 30. RAB17 is a subtype of the Rab GTPase family that can promote M2 polarization through up-regulating PPARγ induced glutamine metabolism 31. The high expression of PPP1R14B might lead to the upregulation of RAC3 and RAB17, further causing to increase the proportion of M2 macrophage to impede the apoptosis of PCa cells. In addition, some immunologic factors are related to regulation of neutrophils. COL17A1 is type XVII collagen that regulates the expression level of the pro-inflammatory chemokine Interleukin-8 (IL-8) that has neutrophil chemotactic activity to inhibit the growth and spread of tumor cells 32, 33. Recent studies in hepatocellular carcinoma showed that PGRMC1, a heme-binding protein, belonged to the membrane-associated progesterone receptor (MAPR) family of cytochrome b5-related proteins 34 and can enhance IL-8 production in multiple immune cells and promotes the recruitment of neutrophils 35. Thus, increased expression of PPP1R14B could cause a reduction in COL17A1 and PGRMC1, which would hinder immune functions of neutrophils. In summary, overexpression of PPP1R14B could result in the upregulation of ALKBH2, UCK2, RAC3 and RAB17 and downregulation of CD40, DKK3, COL17A1 and PGRMC1, which would stifle humoral immunity against PCa through declining the level of plasma cells, block cellular immunity based on NK and CD8+ T cells by upregulating Tregs, destroy cellular immunity according to macrophages via increasing the proportion of M2 macrophages and repress cellular immunity based on neutrophils by means of blocking neutrophil chemotactic activity conclusively inhibiting adaptive immune directed against PCa.

Subsequently, pathway enrichment indicated that the high expression samples of PPP1R14B were mainly related to MYC targets V2, MYC targets V1, transcription of E2F targets under negative control by dream complex, E2F targets, GCM-PFN1, G2M checkpoint, uronic acid metabolic process and so on. PPP1R14B, Protein Phosphatase 1 Regulatory Inhibitor Subunit 14B, is protein phosphatase 1 (PP1) inhibitor that can inhibit the monomer catalytic subunit of PP1, further participate in various phosphorylation dependent signaling pathways, and regulate various cellular processes 36, 37. The enrichment results showed that the overexpression of PPP1R14B is closely related to the body's immunity. The results revealed that the high expression of PPP1R14B is involved in the regulation of MYC and E2F, which are two important transcription factors. Research has shown that PPP1R14B can inhibit PP1, which leads to excessive phosphorylation of MYC on multiple serine and threonine residues, thereby reducing MYC protein levels 36, 38. There is a mutual interaction between MYC and NF-κB 39. In tumor cells, MYC can serve as a positive regulatory factor of NF- κB 40, which is one of the transcription factors of CD40 25. Further, Studies have shown that MYC can reverse regulate the expression of GEFs 41, which is a type of GTPases activator and can activate RAC3 29, 42. Therefore, PPP1R14B can inhibit CD40 and promote RAC3 through PP1/MYC/NF-κB and PP1/MYC/GEFs signaling, respectively, thus suppressing humoral immunity and cellular immunity based on macrophages. Moreover, PP1 can connect PP1 regulatory subunit NIPP1 and PKA to restrain the expression of E2F1 gene 43, which is one of the most important E2F subtypes 44. Research has shown that through differentiation specific marker type 1 transglutaminase (TG-1), E2F can mediate the inhibition of downstream target Sp1, which is a transcription factor for COL17A 45, 46. It can be concluded that PPP1R14B can restrain COL17A through PP1/E2F/SP1 signaling, therefore playing an inhibitory regulatory role in neutrophil mediated cellular immunity. Furthermore, inhibition of PP1 by PPP1R14B can promote the phosphorylation of GSK3β by disrupting the phosphorylation-dephosphorylation balance regulated by AKT2 and PP1, hence activating GSK3β 47, which can inhibit the activation of β-catenin signal 44. β-catenin is a key component of the Wnt signaling pathway, which regulates the transcription of genes such as DKK3 by interacting with transcription factors of the TCF/LEF family 48. Hence, PPP1R14B can inhibit DKK3 through PP1/GSK3β/β-catenin signaling, thus playing a regulatory role on activating Treg cells and further inhibiting CD8+T and NK cell mediated immunity. In summary, PPP1R14B regulates the activity of multiple factors including MYC, E2F and GSK3β by inhibiting the dephosphorylation of PP1, which will adjust the expression of downstream factors such as CD40, RAC3, COL17A, DKK3 and so on. Therefore, overexpression of PPP1R14B can lead to dysregulation of the above signaling, consequently inhibiting adaptive immunity against PCa.

In addition, the enrichment results also showed that the pathways related to the overexpression of PPP1R14B were involved in the proliferation, apoptosis and migration of tumor cells. The dysregulated E2F stimulates the transcription of pro apoptotic and tumor suppressor genes and induces cell apoptosis, thereby protecting cells from tumor invasion 49. E2F1 promotes the invasion and migration of PCa cells by regulating CD147. Overexpression of E2F1 can predict the adverse prognosis of human PCa 50. G2M is an important stage in the cell cycle associated with mitosis. Inhibition of PP1 leads to phosphorylation of Cyclin Dependent Kinases, furtherly inactivating pocket proteins through high phosphorylation and hence promoting cell cycle progression 51. which will stimulate the survival and proliferation of tumor cells 52. Besides, Profilin 1 (PFN1) can adjust the migration of tumor cells, including PCa 53. In conclusion, overexpression of PPP1R14B can promote the proliferation and migration, as well as inhibit apoptosis of PCa cells based on E2F, G2M, PFN1 etc. related pathways.

Recent efforts in cancer therapeutics have focused on the development of targeted therapies directed toward specific molecules related to cancer cell biology to achieve a more specific and effective treatment. Therefore, we evaluated the correlation between hub gene PPP1R14B expression and commonly used drugs sensitivity and observed that patients with higher PPP1R14B expression responded more sensitively to these drugs selumetinib and vorinostat, zebularine, azacitidine and VER155008. The combination of the mitogen-activated protein kinase/extracellular signal-regulated kinase 1/2 inhibitor, selumetinib, and the histone deacetylase inhibitor, vorinostat exerts potent antitumor effects on *in vitro* models of colorectal cancer, including synergistic inhibition of cell proliferation and migration, G1 cell-cycle arrest, induction of apoptosis, and spheroid formation in 3D culture 54. Research suggested that zebularine led to increased levels of interferon response, and promoted infiltration of CD8+T and NK cells into tumor and therefore suppressed tumor growth 55. Effects of azacitidine include direct cytotoxicity from inhibition of protein synthesis and DNA damage and re-expression of aberrantly silenced tumor suppressor genes due to DNA hypomethylation 56, 57. The heat shock protein 70 inhibitor VER155008 suppresses the expression of HSP27, HOP and HSP90β and the androgen receptor, induces apoptosis, and attenuates prostate cancer cell growth 58. This suggests that PPP1R14B may provide a new target for choosing appropriate drugs for PCa patients.

More and more evidence suggests that ceRNA plays a crucial role in cancer. However, there is still a lack of ceRNA networks that can regulate PCa. In this study, we identified 21 miRNAs associated with PPP1R14B, which play important roles in various cancers. Research has found that hsa-miR-6090 is upregulated in PCa patients, which may be related to the occurrence of PCa 59. What's more, has-circ-0044520 and has-circ-0044529 can exist stably in cells for a long time. This stability can block has-miR-4726-5p and has-miR-4640-5p, which can potentially activate downstream oncogenes and initiate the development of laryngeal squamous cell carcinoma 60. It is reported that hsa-miR-134-3p plays a key role in the development of non muscle invasive bladder cancer 61. However, the role of these miRNAs in PCa is still unclear. Furthermore, we also reverse predicted 94 lncRNAs associated with miRNAs, some of which are closely related to PCa. Some studies have shown that LINC00963 promotes the transition of PCa from androgen dependent to androgen independent mode by participating in the trans activation of EGFR 62. More and more evidence suggests that TERC plays an important role in telomere maintenance and other functions in human cancer. Knocking down the TERC by siRNA can reduce proliferation of PCa cell lines. In addition, studies have found a correlation between TERC levels and MYC levels in clinical samples, with forced overexpression of MYC leading to an increase in TERC levels; After MYC silencing, the activity of the human TERC promoter decreases 63. DANCR targeting miR-185-5p via FAK/PI3K/AKT/GSK3 β/snail pathway upregulates LIM and SH3 protein 1, thereby affecting cell migration and proliferation, promoting prostate cancer 64. In addition, it has been reported that HCG11 is abnormally expressed in various tumors and can serve as a key regulatory factor for various cancers such as PCa 65, non-small cell lung cancer 66, and hepatocellular carcinoma 67. The expression level of HCG11 in PCa is significantly reduced, which is directly proportional to the patient's survival rate 68. The ceRNA network indicates the potential regulatory mechanisms of ceRNA on PPP1R14B and PCa. Although some research has been conducted on the regulatory mechanisms in PCa, further exploration is still needed in the future, especially in the ceRNA network.

## Conclusion

Although PPP1R14B may have a certain correlation with the prognosis of uterine corpus endometrial carcinoma, breast cancer and gastrointestinal cancer 69-71, its relationship with PCa has not been studied yet. In our study, hub gene PPP1R14B was screened out through WGCNA, differentially expression analysis, LASSO and COX regression, and had significantly predictive values of diagnosis and prognosis in PCa. Immune and pathway enrichment analysis revealed that overexpression of PPP1R14B could inhibit the dephosphorylation of PP1 to result in the regulation of multiple signaling MYC, E2F, GSK3β and so on in a mess, which accelerated the disorder of several immune- and tumor-related factors, ultimately leading to inhibition of adaptive immunity directed against PCa and promotion of PCa cell proliferation and migration. In summary, our study first proposed that PPP1R14B was a biomarker for evaluating immune characteristics, proliferation and migration in PCa, and had a close relationship with development and prognosis of PCa, which would provide useful insights and a theoretical basis for creating personalized and accurate treatment strategies and drug decisions for patients with PCa.

## Supplementary Material

Supplementary figure.

## Abbreviations

prostate cancer: PCa
metastatic prostate cancer: mPC
metastatic hormone sensitive prostate cancer: mHSPC
androgen deprivation therapy: ADT
metastatic castration resistant prostate cancer: mCRPC
Gene Expression Omnibus: GEO
the Cancer Genome Atlas: TCGA
weighted Gene Co-expression Network Analysis: WGCNA
least absolute shrinkage and selection operator: LASSO
Receiver Operating Characteristic Curve: ROC
Decision Curve Analysis: DCA
competitive endogenous RNA: ceRNA
differentially expressed genes: DEGs
Gene Ontology: GO
cellular components: CCs
molecular functions: MFs
biological pathways: BPs
Kyoto Encyclopedia of Genes and Genomes: KEGG
area under the curve: AUC
Cancer Therapeutics Response Portal: CTRP
half maximal inhibitory concentration: IC50
prostate-specific antigen: PSA
T regulatory cells: Tregs
tumor microenvironment: TME
transforming growth factor β1: TGF-β1
natural killer: NK
transforming growth factor β: TGF β
immunoglobulin: Ig
small guanosine triphosphatases: GTPase
Interleukin-8: IL-8
membrane-associated progesterone receptor: MAPR
protein phosphatase 1: PP1
differentiation specific marker type 1 transglutaminase: TG-1

## References

[1] Siegel RL, Miller KD, Fuchs HE, Jemal A (2021). Cancer Statistics, 2021. CA Cancer J Clin.

[2] Sung H, Ferlay J, Siegel RL, Laversanne M, Soerjomataram I, Jemal A, Bray F (2021). Global Cancer Statistics 2020: GLOBOCAN Estimates of Incidence and Mortality Worldwide for 36 Cancers in 185 Countries. CA Cancer J Clin.

[3] Leech M, Osman S, Jain S, Marignol L (2021). Mini review: Personalization of the radiation therapy management of prostate cancer using MRI-based radiomics. Cancer Lett.

[4] DE Nunzio C, Fiori C, Fusco F, Gregori A, Pagliarulo V, Alongi F (2022). Androgen deprivation therapy and cardiovascular risk in prostate cancer. Minerva Urol Nephrol.

[5] Gheorghe GS, Hodorogea AS, Ciobanu A, Nanea IT, Gheorghe ACD (2021). Androgen Deprivation Therapy, Hypogonadism and Cardiovascular Toxicity in Men with Advanced Prostate Cancer. Curr Oncol.

[6] Barata PC, Sartor AO (2019). Metastatic castration-sensitive prostate cancer: Abiraterone, docetaxel, or…. Cancer.

[7] Ritch C, Cookson M (2018). Recent trends in the management of advanced prostate cancer. F1000Res.

[8] Buck SAJ, Koolen SLW, Mathijssen RHJ, de Wit R, van Soest RJ (2021). Cross-resistance and drug sequence in prostate cancer. Drug Resist Updat.

[9] Wang G, Zhao D, Spring DJ, DePinho RA (2018). Genetics and biology of prostate cancer. Genes Dev.

[10] Kolluri S, Lin J, Liu R, Zhang Y, Zhang W (2022). Machine Learning and Artificial Intelligence in Pharmaceutical Research and Development: a Review. AAPS J.

[11] Ferlay J, Soerjomataram I, Dikshit R, Eser S, Mathers C, Rebelo M, Parkin DM, Forman D, Bray F (2015). Cancer incidence and mortality worldwide: sources, methods and major patterns in GLOBOCAN 2012. Int J Cancer.

[12] Drake RR, Angel PM, Wu J, Pachynski RK, Ippolito JE (2020). How else can we approach prostate cancer biomarker discovery?. Expert Rev Mol Diagn.

[13] Li D, Xu W, Chang Y, Xiao Y, He Y, Ren S (2023). Advances in landscape and related therapeutic targets of the prostate tumor microenvironment. Acta Biochim Biophys Sin (Shanghai).

[14] Bansal A, Simon MC (2018). Glutathione metabolism in cancer progression and treatment resistance. J Cell Biol.

[15] Lin X, Zhuang S, Chen X, Du J, Zhong L, Ding J, Wang L, Yi J, Hu G, Tang G, Luo X, Liu W, Ye F (2022). lncRNA ITGB8-AS1 functions as a ceRNA to promote colorectal cancer growth and migration through integrin-mediated focal adhesion signaling. Mol Ther.

[16] Rizzo A, Santoni M, Mollica V, Fiorentino M, Brandi G, Massari F (2022). Microbiota and prostate cancer. Semin Cancer Biol.

[17] Zhang LS, Chen QC, Zong HT, Xia Q (2023). Exosome miRNA-203 promotes M1 macrophage polarization and inhibits prostate cancer tumor progression. Mol Cell Biochem.

[18] Tanaka A, Sakaguchi S (2017). Regulatory T cells in cancer immunotherapy. Cell Res.

[19] Farhood B, Najafi M, Mortezaee K (2019). CD8+ cytotoxic T lymphocytes in cancer immunotherapy: A review. J Cell Physiol.

[20] Kanamori M, Nakatsukasa H, Okada M, Lu Q, Yoshimura A (2016). Induced Regulatory T Cells: Their Development, Stability, and Applications. Trends Immunol.

[21] Nutt SL, Hodgkin PD, Tarlinton DM, Corcoran LM (2015). The generation of antibody-secreting plasma cells. Nat Rev Immunol.

[22] Elgueta R, Benson MJ, de Vries VC, Wasiuk A, Guo Y, Noelle RJ (2009). Molecular mechanism and function of CD40/CD40L engagement in the immune system. Immunol Rev.

[23] Danese S, Sans M, Fiocchi C (2004). The CD40/CD40L costimulatory pathway in inflammatory bowel disease. Gut.

[24] Ke B, Ye K, Cheng S (2020). ALKBH2 inhibition alleviates malignancy in colorectal cancer by regulating BMI1-mediated activation of NF-κB pathway. World J Surg Oncol.

[25] Kojok K, Akoum SE, Mohsen M, Mourad W, Merhi Y (2018). CD40L Priming of Platelets via NF-κB Activation is CD40- and TAK1-Dependent. J Am Heart Assoc.

[26] Cahill MA, Jazayeri JA, Catalano SM, Toyokuni S, Kovacevic Z, Richardson DR (2016). The emerging role of progesterone receptor membrane component 1 (PGRMC1) in cancer biology. Biochim Biophys Acta.

[27] Liu DH, Wen GM, Song CL, Xia P (2023). Effect of secretory DKK3 on circulating CD56 natural killer cells in patients with liver cancer. Int J Biol Markers.

[28] Al Shareef Z, Kardooni H, Murillo-Garzón V, Domenici G, Stylianakis E, Steel JH, Rabano M, Gorroño-Etxebarria I, Zabalza I, Vivanco MD, Waxman J, Kypta RM (2018). Protective effect of stromal Dickkopf-3 in prostate cancer: opposing roles for TGFBI and ECM-1. Oncogene.

[29] Liu TQ, Wang GB, Li ZJ, Tong XD, Liu HX (2015). Silencing of Rac3 inhibits proliferation and induces apoptosis of human lung cancer cells. Asian Pac J Cancer Prev.

[30] Travnickova J, Nhim S, Abdellaoui N, Djouad F, Nguyen-Chi M, Parmeggiani A, Kissa K (2021). Macrophage morphological plasticity and migration is Rac signalling and MMP9 dependant. Sci Rep.

[31] Zhang X, Chen C, Ling C, Luo S, Xiong Z, Liu X, Liao C, Xie P, Liu Y, Zhang L, Chen Z, Liu Z, Tang J (2022). EGFR tyrosine kinase activity and Rab GTPases coordinate EGFR trafficking to regulate macrophage activation in sepsis. Cell Death Dis.

[32] Van den Bergh F, Eliason SL, Burmeister BT, Giudice GJ (2012). Collagen XVII (BP180) modulates keratinocyte expression of the proinflammatory chemokine, IL-8. Exp Dermatol.

[33] Matsushima K, Yang D, Oppenheim JJ (2022). Interleukin-8: An evolving chemokine. Cytokine.

[34] Wu D, Zhang C, Liao G, Leng K, Dong B, Yu Y, Tai H, Huang L, Luo F, Zhang B, Zhan T, Hu Q, Tai S (2022). Targeting uridine-cytidine kinase 2 induced cell cycle arrest through dual mechanism and could improve the immune response of hepatocellular carcinoma. Cell Mol Biol Lett.

[35] Dumitru CA, Schröder H, Schäfer FTA, Aust JF, Kreße N, Siebert CLR, Stein KP, Haghikia A, Wilkens L, Mawrin C, Sandalcioglu IE (2023). Progesterone Receptor Membrane Component 1 (PGRMC1) Modulates Tumour Progression, the Immune Microenvironment and the Response to Therapy in Glioblastoma. Cells.

[36] Deng M, Peng L, Li J, Liu X, Xia X, Li G (2021). PPP1R14B Is a Prognostic and Immunological Biomarker in Pan-Cancer. Front Genet.

[37] Xiang N, Chen T, Zhao X, Zhao M (2022). In vitro assessment of roles of PPP1R14B in cervical and endometrial cancer. Tissue Cell.

[38] Dingar D, Tu WB, Resetca D, Lourenco C, Tamachi A, De Melo J, Houlahan KE, Kalkat M, Chan PK, Boutros PC, Raught B, Penn LZ (2018). MYC dephosphorylation by the PP1/PNUTS phosphatase complex regulates chromatin binding and protein stability. Nat Commun.

[39] Zhang L, Shao G, Shao J, Zhao J (2022). PRMT5-activated c-Myc promote bladder cancer proliferation and invasion through up-regulating NF-κB pathway. Tissue Cell.

[40] Marquardt V, Theruvath J, Pauck D, Picard D, Qin N, Blümel L, Maue M, Bartl J, Ahmadov U, Langini M, Meyer FD, Cole A, Cruz-Cruz J, Graef CM, Wölfl M, Milde T, Witt O, Erdreich-Epstein A, Leprivier G, Kahlert U, Stefanski A, Stühler K, Keir ST, Bigner DD, Hauer J, Beez T, Knobbe-Thomsen CB, Fischer U, Felsberg J, Hansen FK, Vibhakar R, Venkatraman S, Cheshier SH, Reifenberger G, Borkhardt A, Kurz T, Remke M, Mitra S (2023). Tacedinaline (CI-994), a class I HDAC inhibitor, targets intrinsic tumor growth and leptomeningeal dissemination in MYC-driven medulloblastoma while making them susceptible to anti-CD47-induced macrophage phagocytosis via NF-kB-TGM2 driven tumor inflammation. J Immunother Cancer.

[41] Zeng RJ, Xie WJ, Zheng CW, Chen WX, Wang SM, Li Z, Cheng CB, Zou HY, Xu LY, Li EM (2021). Role of Rho guanine nucleotide exchange factors in non-small cell lung cancer. Bioengineered.

[42] Zamboni V, Jones R, Umbach A, Ammoni A, Passafaro M, Hirsch E, Merlo GR (2018). Rho GTPases in Intellectual Disability: From Genetics to Therapeutic Opportunities. Int J Mol Sci.

[43] Hanaki S, Habara M, Masaki T, Maeda K, Sato Y, Nakanishi M, Shimada M (2021). PP1 regulatory subunit NIPP1 regulates transcription of E2F1 target genes following DNA damage. Cancer Sci.

[44] Gao S, Wang S, Zhao Z, Zhang C, Liu Z, Ye P, Xu Z, Yi B, Jiao K, Naik GA, Wei S, Rais-Bahrami S, Bae S, Yang WH, Sonpavde G, Liu R, Wang L (2022). TUBB4A interacts with MYH9 to protect the nucleus during cell migration and promotes prostate cancer via GSK3β/β-catenin signalling. Nat Commun.

[45] van Zalen S, Nijenhuis M, Jonkman MF, Pas HH (2006). Two major 5'-untranslated regions for type XVII collagen mRNA. J Dermatol Sci.

[46] Wong CF, Barnes LM, Dahler AL, Smith L, Popa C, Serewko-Auret MM, Saunders NA (2005). E2F suppression and Sp1 overexpression are sufficient to induce the differentiation-specific marker, transglutaminase type 1, in a squamous cell carcinoma cell line. Oncogene.

[47] Yadav Y, Sharma M, Dey CS (2023). PP1γ regulates neuronal insulin signaling and aggravates insulin resistance leading to AD-like phenotypes. Cell Commun Signal.

[48] Baetta R, Banfi C (2019). Dkk (Dickkopf) Proteins. Arterioscler Thromb Vasc Biol.

[49] Nakajima R, Zhao L, Zhou Y, Shirasawa M, Uchida A, Murakawa H, Fikriyanti M, Iwanaga R, Bradford AP, Araki K, Warita T, Ohtani K (2023). Deregulated E2F Activity as a Cancer-Cell Specific Therapeutic Tool. Genes (Basel).

[50] Liang YX, Lu JM, Mo RJ, He HC, Xie J, Jiang FN, Lin ZY, Chen YR, Wu YD, Luo HW, Luo Z, Zhong WD (2016). E2F1 promotes tumor cell invasion and migration through regulating CD147 in prostate cancer. Int J Oncol.

[51] Kurimchak A, Graña X (2012). PP2A holoenzymes negatively and positively regulate cell cycle progression by dephosphorylating pocket proteins and multiple CDK substrates. Gene.

[52] Sun Y, Liu Y, Ma X, Hu H (2021). The Influence of Cell Cycle Regulation on Chemotherapy. Int J Mol Sci.

[53] Pečar Fonović U, Kos J (2015). Cathepsin X Cleaves Profilin 1 C-Terminal Tyr139 and Influences Clathrin-Mediated Endocytosis. PLoS One.

[54] Hedayat M, Jafari R, Majidi Zolbanin N (2023). Selumetinib: a selective MEK1 inhibitor for solid tumor treatment. Clin Exp Med.

[55] Lai J, Fu Y, Tian S, Huang S, Luo X, Lin L, Zhang X, Wang H, Lin Z, Zhao H, Lin S, Zhao J, Xu S, Li D, Cai S, Dong L, Qian J, Liang J, Li Q, Zhang Y, Fan J, Balderas R, Chen Q (2021). Zebularine elevates STING expression and enhances cGAMP cancer immunotherapy in mice. Mol Ther.

[56] Hollenbach PW, Nguyen AN, Brady H, Williams M, Ning Y, Richard N, Krushel L, Aukerman SL, Heise C, MacBeth KJ (2010). A comparison of azacitidine and decitabine activities in acute myeloid leukemia cell lines. PLoS One.

[57] Vatanmakanian M, Steffan JJ, Koul S, Ochoa AC, Chaturvedi LS, Koul HK (2023). Regulation of SPDEF expression by DNA methylation in advanced prostate cancer. Front Endocrinol (Lausanne).

[58] Brünnert D, Langer C, Zimmermann L, Bargou RC, Burchardt M, Chatterjee M, Stope MB (2020). The heat shock protein 70 inhibitor VER155008 suppresses the expression of HSP27, HOP and HSP90β and the androgen receptor, induces apoptosis, and attenuates prostate cancer cell growth. J Cell Biochem.

[59] Kim J, Shim JS, Han BH, Kim HJ, Park J, Cho IJ, Kang SG, Kang JY, Bong KW, Choi N (2021). Hydrogel-based hybridization chain reaction (HCR) for detection of urinary exosomal miRNAs as a diagnostic tool of prostate cancer. Biosens Bioelectron.

[60] Fan Y, Xia X, Zhu Y, Diao W, Zhu X, Gao Z, Chen X (2018). Circular RNA Expression Profile in Laryngeal Squamous Cell Carcinoma Revealed by Microarray. Cell Physiol Biochem.

[61] He YH, Deng YS, Peng PX, Wang N, Wang JF, Ding ZS, Chen X, Zhou XF (2019). A novel messenger RNA and long noncoding RNA signature associated with the progression of nonmuscle invasive bladder cancer. J Cell Biochem.

[62] Xie Z, Zhong C, Shen J, Jia Y, Duan S (2022). LINC00963: A potential cancer diagnostic and therapeutic target. Biomed Pharmacother.

[63] Baena-Del Valle JA, Zheng Q, Esopi DM, Rubenstein M, Hubbard GK, Moncaliano MC, Hruszkewycz A, Vaghasia A, Yegnasubramanian S, Wheelan SJ, Meeker AK, Heaphy CM, Graham MK, De Marzo AM (2018). MYC drives overexpression of telomerase RNA (hTR/TERC) in prostate cancer. J Pathol.

[64] Sun W, Zu S, Shao G, Wang W, Gong F (2021). Long non-coding DANCR targets miR-185-5p to upregulate LIM and SH3 protein 1 promoting prostate cancer via the FAK/PI3K/AKT/GSK3β/snail pathway. J Gene Med.

[65] Wang YC, He WY, Dong CH, Pei L, Ma YL (2019). lncRNA HCG11 regulates cell progression by targeting miR-543 and regulating AKT/mTOR pathway in prostate cancer. Cell Biol Int.

[66] Fan G, Jiao J, Shen F, Ren Q, Wang Q, Chu F (2020). Long non-coding RNA HCG11 sponging miR-522-3p inhibits the tumorigenesis of non-small cell lung cancer by upregulating SOCS5. Thorac Cancer.

[67] Li ML, Zhang Y, Ma LT (2019). LncRNA HCG11 accelerates the progression of hepatocellular carcinoma via miR-26a-5p/ATG12 axis. Eur Rev Med Pharmacol Sci.

[68] Yuan X, Zhao Q, Zhang Y, Xue M (2021). The role and mechanism of HLA complex group 11 in cancer. Biomed Pharmacother.

[69] He K, Wang T, Huang X, Yang Z, Wang Z, Zhang S, Sui X, Jiang J, Zhao L (2023). PPP1R14B is a diagnostic prognostic marker in patients with uterine corpus endometrial carcinoma. J Cell Mol Med.

[70] Huang P, Xia L, Guo Q, Huang C, Wang Z, Huang Y, Qin S, Leng W, Li D (2022). Genome-wide association studies identify miRNA-194 as a prognostic biomarker for gastrointestinal cancer by targeting ATP6V1F, PPP1R14B, BTF3L4 and SLC7A5. Front Oncol.

[71] Zhou L, Zhang L, Guan X, Dong YI, Liu T (2022). Long noncoding RNA PPP1R14B-AS1 imitates microRNA-134-3p to facilitate breast cancer progression by upregulating LIM and SH3 protein 1. Oncol Res.

