# Supplementary figures and images for: PPP1R14B as a potential biomarker for the identification of diagnosis and prognosis affecting tumor immunity, proliferation and migration in prostate cancer
Source: J Cancer. 2024 Oct 21;15(20):6545–64. doi: 10.7150/jca.101100 (PMC11632978; doi:10.7150/jca.101100)

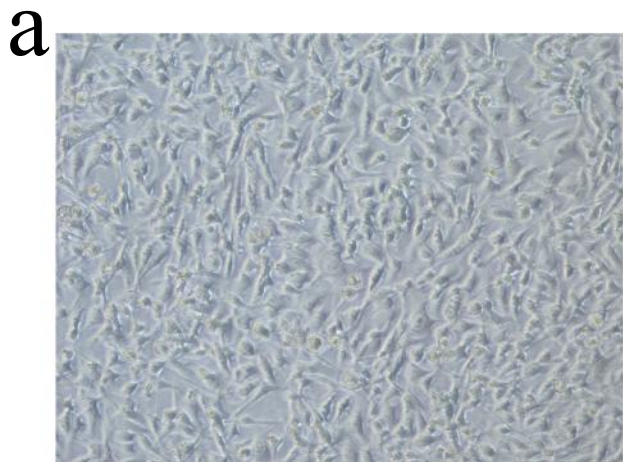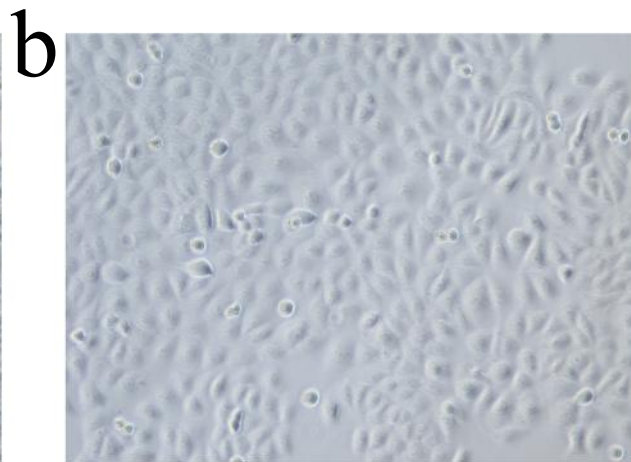

**Supplementary Fig. 1** cells image. **a** PC-3, **b** RWPE-1.

Supplement: Supplementary file 1 — Supplementary figure. [file jcav15p6545s1.pdf]
